# P-1318. Antimicrobial Activity of Aztreonam-Avibactam and Comparator Agents against Stenotrophomonas maltophilia Isolates from Patients Hospitalized with Pneumonia in Europe, Latina America, and Asia (2020-2024)

**DOI:** 10.1093/ofid/ofaf695.1506

**Published:** 2026-01-11

**Authors:** Helio SaderMariana Castanheira, Gregory Stone, Katherine Perez, Marisa Winkler, Rodrigo E Mendes

**Affiliations:** Element, North Liberty, IA; Pfizer, Inc., Groton, Connecticut; Pfizer, New York, New York; Element Materials Technology/Jones Microbiology Institute, North Liberty, Iowa; Element Iowa City (JMI Laboratories), North Liberty, IA

## Abstract

**Background:**

Aztreonam-avibactam (ATM-AVI) has recently been approved for clinical use in the European Union and United States to treat Enterobacterales infections, including those caused by metallo-β-lactamase (MBL) producers. We evaluated the activity of ATM-AVI against *S. maltophilia* isolated from patients with pneumonia.

**Table 1.**
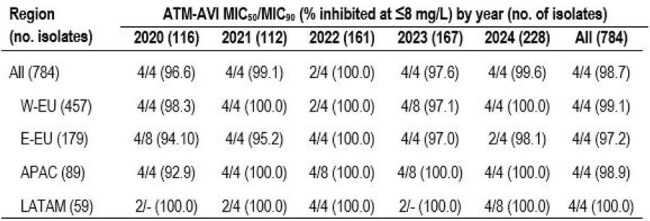
Aztreonam-avibactam (ATM-AVI) activity stratified by region and year Abbreviations: W-EU, Western Europe; E-EU, Eastern Europe; APAC, Asia Pacific region; LATAM, Latin America.

**Figure:**
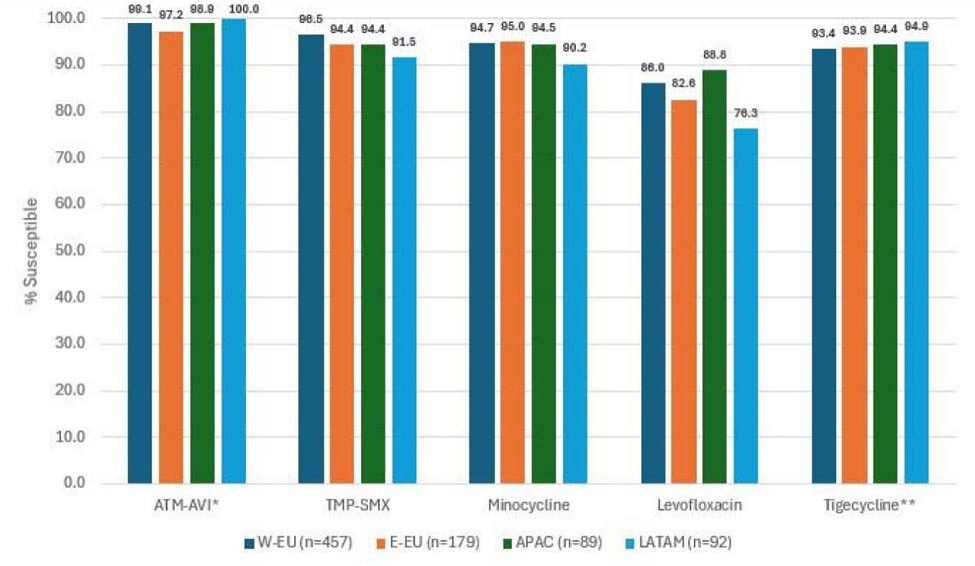
Antimicrobial activity of aztreonam-avibactam (ATM-AVI) and comparators against S. maltophilia isolates from patients with pneumonia stratified by region * % inhibited at ≤8 mg/L. ** % inhibited at ≤2 mg/L. Abbreviations: ATM-AVI, aztreonam-avibactam; TMP-SMX, trimethoprim-sulfamethoxazole; W-EU, Western Europe; E-EU, Eastern Europe, APAC, Asia-Pacific region; LATAM; Latin America.

**Methods:**

784 clinical isolates were collected from 60 medical centers located in Western Europe (W-EU; n=457; 25 centers in 10 countries), Eastern Europe (E-EU; n=179; 16 centers in 9 countries), the Asia-Pacific region (APAC; n=89; 10 centers in 5 countries), and Latin America (LATAM; n=59; 9 centers in 6 countries), and as part of the SENTRY Antimicrobial Surveillance Program. Isolates were susceptibility tested by CLSI M07 broth microdilution method. An ATM-AVI pharmacodynamic/pharmacokinetic (PK/PD) susceptible breakpoint of ≤ 8 mg/L was applied for comparison. CLSI breakpoints were applied to comparators when available.

**Figure:**
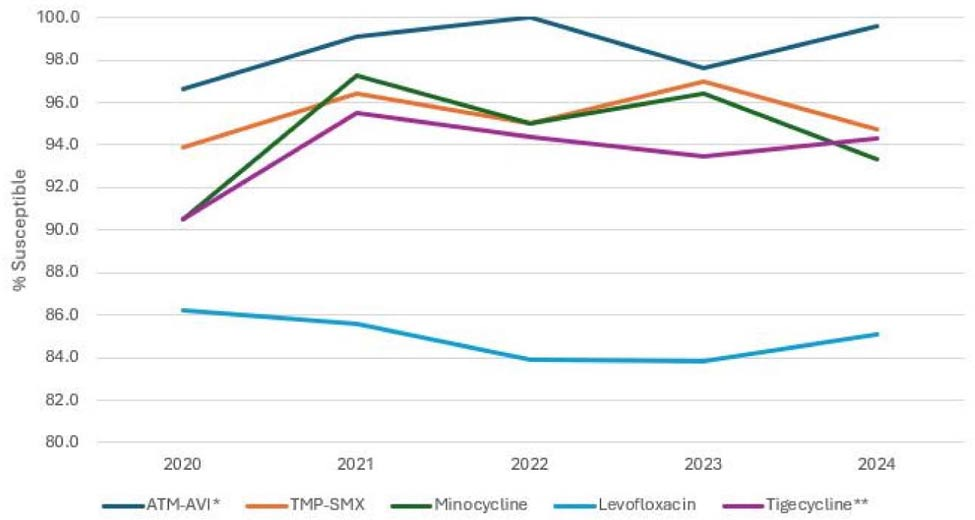
Antimicrobial activity of aztreonam-avibactam (ATM-AVI) and comparators against S. maltophilia isolates from patients with pneumonia stratified by year * % inhibited at ≤8 mg/L. ** % inhibited at ≤2 mg/L. Abbreviations: ATM-AVI, aztreonam-avibactam; TMP-SMX, trimethoprim-sulfamethoxazole.

**Figure:**
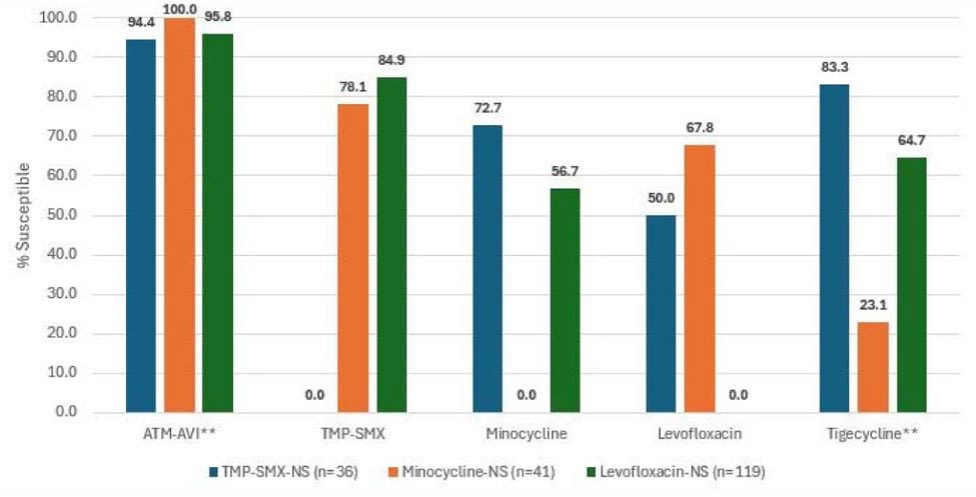
Antimicrobial activity of aztreonam-avibactam (ATM-AVI) and comparators against resistant subsets * % inhibited at ≤8 mg/L. ** % inhibited at ≤2 mg/L. Abbreviations: ATM-AVI, aztreonam-avibactam; TMP-SMX, trimethoprim-sulfamethoxazole; NS, not susceptible.

**Results:**

ATM-AVI demonstrated potent activity against isolates from all geographic regions (overall MIC_50/90_, 4/4 mg/L; 98.7% inhibited at ≤ 8 mg/L), and its activity remained stable during the study period (Table 1 and Figures 1 and 2). The percentage inhibited at ≤8 mg/L ranged from 97.2% in E-EU to 100.0% in LATAM. Trimethoprim-sulfamethoxazole (TMP-SMX; MIC_50/90_, ≤ 0.12/0.5 mg/L; 95.4% susceptible) and minocycline (MIC_50/90_, 0.5/1 mg/L; 94.5% susceptible) also were very active. Levofloxacin was active against 84.8% of isolates per CLSI criteria. Ceftazidime (MIC_50/90_, > 32/ > 32 mg/L; 13.5% inhibited at ≤ 8 mg/L) and colistin (MIC_50/90_, 4/ > 8 mg/L; 38.8% inhibited at ≤ 2 mg/L) showed limited activity. ATM-AVI retained potent activity against isolates not susceptible to TMP-SMX, minocycline, and/or levofloxacin (Figure 3).

**Conclusion:**

Our results indicated that ATM-AVI may represent a valuable option to treat *S. maltophilia* infections, addressing a major unmet medical need. *S. maltophilia* susceptibility rates for comparator agents should be interpreted with caution since breakpoints were established in the 1980s; i.e. before the knowledge of PK/PD parameters that are currently used to establish breakpoints.

**Disclosures:**

Helio Sader, United States Food and Drug Administration: FDA Contract Number: 75F40123C00140 Mariana Castanheira, PhD, Melinta Therapeutics: Advisor/Consultant|Melinta Therapeutics: Grant/Research Support Marisa Winkler, MD, PhD, Basilea: Advisor/Consultant|Basilea: Grant/Research Support|GSK: Advisor/Consultant|GSK: Grant/Research Support|Melinta Therapeutics: Advisor/Consultant|Melinta Therapeutics: Grant/Research Support|Mundipharma: Advisor/Consultant|Mundipharma: Grant/Research Support|Pfizer: Advisor/Consultant|Pfizer: Grant/Research Support|Pulmocide: Advisor/Consultant|Pulmocide: Grant/Research Support Rodrigo E. Mendes, PhD, GSK: Grant/Research Support|Shionogi & Co., Ltd.: Grant/Research Support|United States Food and Drug Administration: FDA Contract Number: 75F40123C00140

